# Inpatient burden of respiratory syncytial virus (RSV) in Switzerland, 2003 to 2021: an analysis of administrative data

**DOI:** 10.2807/1560-7917.ES.2024.29.39.2400119

**Published:** 2024-09-26

**Authors:** Michael Stucki, Golda Lenzin, Philipp KA Agyeman, Klara M Posfay-Barbe, Nicole Ritz, Johannes Trück, Angela Fallegger, Susanne G Oberle, Oliver Martyn, Simon Wieser

**Affiliations:** 1 ZHAW Zurich University of Applied Sciences, School of Management and Law, Winterthur Institute of Health Economics, Winterthur, Switzerland; 2 Division of Pediatric Infectious Disease, Department of Pediatrics, Inselspital, Bern University Hospital, University of Bern, Bern, Switzerland; 3 Department of Pediatrics, Gynecology and Obstetrics, Geneva University Hospitals and Faculty of Medicine, University of Geneva, Geneva, Switzerland; 4 Children’s Hospital of Central Switzerland, Department of Paediatrics and Infectious Diseases Unit and Faculty of Health Science and Medicine, University of Lucerne, Lucerne, Switzerland; 5 Mycobacterial and Migrant Health Research, University Children’s Hospital Basel and Department for Clinical Research, University of Basel, Basel, Switzerland; 6 Divisions of Allergy and Immunology, University Children’s Hospital and Children’s Research Center, University of Zurich (UZH), Zurich, Switzerland; 7 Sanofi AG, Vaccines Medical Affairs, Rotkreuz, Switzerland; 8 Sanofi A/S, Vaccines Medical Affairs, Copenhagen, Denmark

**Keywords:** Switzerland, respiratory syncytial virus, inpatient care, administrative data, burden, epidemiology, health care resource use, infants, children, adults

## Abstract

**Background:**

Respiratory syncytial virus (RSV) is a leading cause of acute respiratory infections and hospitalisations in infants (age < 1 year) and young children. Little is known on RSV epidemiology and related inpatient healthcare resource use (HCRU) in Switzerland.

**Aim:**

To explore RSV-related hospitalisations, inpatient HCRU and medical costs in all age groups, and risk factors for infant hospitalisations in Switzerland.

**Methods:**

We used national hospital registry data from 2003 to 2021 identifying RSV cases with ICD-10-GM codes, and described demographic characteristics, HCRU and associated medical costs of RSV inpatients. The effect of risk factors on infant hospitalisation was estimated with logistic regression.

**Results:**

We observed a general increase and biannual pattern in RSV hospitalisations between 2003/04 and 2018/19, with 3,575 hospitalisations in 2018/19 and 2,487 in 2019/20 before numbers declined in 2020/21 (n = 902). Around two thirds of all hospitalisations occurred in infants. Mean (median) age was 118 (85) days in hospitalised infants and 74 (77) years in hospitalised adult patients (> 18 years); 7.2% of cases required intensive care unit stay. Mean inpatient medical costs were estimated at EUR 8,046. Most (90.8%) hospitalised infants with RSV were born after 35 weeks of gestation without bronchopulmonary dysplasia or congenital heart disease. Low birth weight, gestational age and congenital disorders were associated with a higher risk for hospitalisation.

**Conclusions:**

RSV leads to a substantial number of hospitalisations and peaks in hospital capacity utilisation. Measures to protect all infants from an RSV hospitalisation are essential in addressing this public health challenge.

**Key public health message**

**What did you want to address in this study and why?**

Respiratory syncytial virus (RSV) causes acute respiratory infections and leads to many hospitalisations in children. Because cases of RSV do not need to be reported, there is not much evidence on the long-term epidemiology of RSV in Switzerland. We aimed to describe the burden of RSV in inpatient care using administrative data from Swiss hospitals.

**What have we learnt from this study?**

The number of RSV hospitalisations followed a biannual pattern. Hospitalisations increased between 2003 and 2020 before numbers fell substantially in the winter of 2020/21 during the COVID-19 pandemic. About two thirds of all RSV hospitalisations occurred in infants under 1 year. Most of these infants were previously healthy and without any medical risk factor. One inpatient stay for RSV costs about 8,000 EUR on average.

**What are the implications of your findings for public health?**

Respiratory syncytial virus causes a high number of hospitalisations in infants and young children and results in substantial peaks in hospital capacity utilisation. Our study shows that protective measures against RSV hospitalisations are essential.

## Introduction

Respiratory syncytial virus (RSV) is the leading cause of acute respiratory infections (ARI) and a major cause of hospitalisations in infants (aged < 1 year) and young children worldwide [1,2]. A recent review showed that 12–63% of all ARI and 19–81% of viral ARI that led to a hospitalisation in Western countries were linked to RSV [1]. Most hospitalisations caused by RSV are observed in infants under 6 months of age [2]. Numerous studies have identified risk factors for RSV infections and/or RSV-associated hospitalisations in infants or children [3-6]. Newborns with certain congenital disorders and those born prematurely have a substantially higher risk of complication from an RSV infection. Nevertheless, most hospitalisations occur in healthy children born at term and without any pre-existing medical conditions known to increase the risk of hospitalisation [7,8].

Respiratory syncytial virus incidence in healthy term born infants born between 2017 and 2020 in Europe was estimated at 26.2%, and RSV-related hospitalisations at 1.8% [9]. Surveillance for RSV is not well established in Switzerland and the disease is not notifiable. Thus, the introduction of the RSV EpiCH hospital surveillance network in 2021 is a promising development [10]. However, data for RSV EpiCH are collected on a voluntary basis and does not cover all Swiss hospitals. One of the few studies from Switzerland with data on children hospitalised for RSV between 2001 and 2005 estimated that ca 1,000 infants – equivalent to around 1.36% of the annual birth cohort – were admitted with RSV in Switzerland annually [11]. The pattern of hospitalisations was characterised by a biannual variation [12].

Despite RSV infections being frequent, little is known about the burden of hospitalisations and healthcare resources utilisation (HCRU) associated with RSV in Switzerland. Analysing and summarising national data on age-specific hospital (inpatient-treated) incidence and hospitalisation rates, HCRU, medical costs and patient characteristics is critical to inform about the inpatient burden of disease in Switzerland and to provide inputs for health economic analyses. Moreover, the evaluation of risk factors for RSV hospitalisations in infants may help to identify newborns most at risk of severe infection, aiding in targeted intervention strategies and preventive measures. The need for a systematic monitoring has further increased with the substantial change of RSV epidemiology during the COVID-19 pandemic. Studies from Switzerland and other countries found fewer cases during typical RSV seasons than in previous years [10,13-15]. This study aims to describe the long-term RSV epidemiology in inpatient care and the characteristics and the HCRU of RSV inpatients in Switzerland using data from the national hospital inpatient registry. Furthermore, it assesses the risk factors for RSV hospitalisations in infants. 

## Methods

### Study setting

We used administrative anonymised case-level data from the Swiss hospital inpatient registry (HospReg; Medizinische Statistik der Krankenhäuser) [16]. HospReg is produced by the Federal Statistical Office (FSO) since 1998 and covers all inpatient stays in Swiss hospitals, i.e. from all cantons. It holds detailed information on diagnoses, procedures, in-hospital deaths, length of stay (LOS), month of admission, as well as age and sex of hospitalised patients. HospReg holds additional variables for newborns such as gestational age, birth weight and mother’s age at birth. Importantly, HospReg allows tracking patients over time and across public and private hospitals.

We included all inpatients treated for RSV across all age groups, with a special focus on infants and young children. Data on adult RSV inpatients were included to contextualise results for the paediatric population.

### Case definition and classification

Hospitalisations for RSV, other ARI and other medical conditions were identified using main and secondary diagnoses according to the International Classification of Diseases (ICD), 10th revision, German modification (ICD-10-GM) [17] available for each inpatient stay. An ‘RSV*’* case was identified as a hospitalisation with one of the following codes as main or secondary diagnosis: J21.0 (acute bronchiolitis due to RSV), J12.1 (RSV pneumonia), J20.5 (acute bronchitis due to RSV) and B97.4 (RSV as the cause of diseases classified to other chapters). ‘Other ARI’ cases include the codes J00–J06 (acute upper respiratory infections), J09–J18 (influenza and pneumonia) and J20–J22 (other acute lower respiratory infection) – excluding RSV codes. In Switzerland, RSV is mostly diagnosed using PCR testing (usually multiplex PCR).

### Analyses

We performed two analyses. Firstly, we performed a descriptive analysis of the number of paediatric (< 18 years) and adult (≥ 18 years) hospitalised RSV cases (2003–21), the associated hospitalisation rates for the three most recent respiratory years (2018/19 to 2020/21) as well as the characteristics of infant RSV inpatients and the resource use and medical costs in all RSV inpatients for the 6 most recent years (2016–21). 

Secondly, we used multivariate logistic regression to estimate the effect of risk factors on RSV hospitalisation in the first year of life. For this analysis, we used data on all children born in Swiss hospitals between 2012 and 2020. 

We chose different periods for the analyses because we wanted to exploit our rich dataset and show the long-term trend in RSV hospitalisations (starting from 2003), while at the same time ensuring a sufficient quality of diagnostic coding (starting from 2012) and up-to-date estimates of hospitalisation rates and HCRU (starting from 2016). Since 2012, HospReg is used for the calculation of diagnosis-related groups (DRG) cost weights. This has led to a substantial increase in coding quality. Based on information from the FSO and on our own experience with previous datasets, we considered coding quality sufficiently reliable from 2003 on, and very high from 2012 on.

RSV respiratory years were defined as the period from July to June the following year. Furthermore, we analysed the data stratified by age groups (0–30 days; 31–90 days; 91–180 days; 181–364 days; 1 year; 2–4 years; 5–9 years; 10–19 years; 20–39 years; 40–59 years; 60–79 years; ≥ 80 years).

#### Hospitalisation numbers and rates in paediatric and adult patients

We estimated hospitalisation rates, defined as the number of hospitalisations with an RSV main diagnosis in 1,000 population by age groups and RSV respiratory year. Population numbers stem from FSO population statistics [18]. We used the end-of-the-year population as an estimate of the population in each RSV respiratory year, e.g. population by 31 December 2018 for RSV respiratory year 2018/19. The size of the Swiss population was 8.7 million in 2021 increasing from 8.0 million in 2012. The population statistics did not report the number of individuals in more granular age groups below 1 year of age. We estimated the population for these age groups by dividing the total number of live births as reported by the FSO [19] by 12, assuming an equal distribution of births in each month. This method has been applied in previous research [20,21].

#### RSV-associated inpatient healthcare resource use and medical costs in paediatric and adult patients

For each case, HospReg records the number of hours admitted to the intensive care unit (ICU) (allowing for identification of patients treated in ICU) and the number of ventilation hours. We reported the average LOS of cases with an RSV main diagnosis, the rate of RSV cases requiring ICU treatment and/or ventilation (including non-invasive ventilation outside of ICU) as well as the mean number of hours in ICU and with ventilation by age group.

For the calculation of RSV-related medical costs in inpatient care, we used the SwissDRG online batch grouper (based on the SwissDRG catalogue [22]) to obtain cost weights of all inpatient cases with an RSV main diagnosis as well as cases with an ARI main diagnosis and RSV secondary diagnosis. Medical costs per patient were determined by multiplying the DRG cost weight with a hospital-specific base rate. Base rates are not included in HospReg data. We assumed an average base rate of Swiss Francs (CHF) 10,000 (EUR 9,251), corresponding approximately to the average base rate in the canton of Zurich, the largest Swiss canton, between 2012 and 2021 [23]. We converted all estimates in Swiss Francs (CHF) to Euro (EUR) using the annual average of the CHF/EUR exchange rate in 2021 as provided by the Swiss national bank [24].

#### Proportion of RSV and other acute respiratory infection hospitalisations

We analysed the proportion of RSV, other ARI, and other main diagnoses (excluding RSV and other ARI) of all hospitalisations between 2003 and 2021, based on monthly hospitalisations, in total and for infants only.

#### Risk factors for RSV hospitalisation in infants

We performed multivariate logistic regression analysis to assess the effect of established risk factors on the probability of being admitted to hospital with an RSV main or secondary diagnosis as an infant, i.e. in the first year of life. The list of risk factors was created based on current available evidence by the study team. Risk factors were either birth-related (year and quarter of birth, birth weight, gestational age, an indicator for multiple birth, age of mother at birth, sex) or medical diagnoses (e.g. Down’s syndrome). We identified medical conditions based on ICD-10-GM codes. Medical conditions were only included if there was a statistically significant difference between the RSV and the non-RSV populations in a univariate comparison (applying a p value of < 0.05 derived from a Z-test statistic for proportions).

We used data for all children born in Swiss hospitals between 2012 and 2020. There were on average 85,771 live births per year between 2012 and 2020, ranging from 82,164 in 2012 to 87,883 in 2016 [19]. Based on HospReg data, around 99.5% of these births took place in hospitals. Births before 2012 were excluded from the risk factor analysis because of likely undercoding of RSV before DRG implementation. Births in 2021 were excluded because our dataset did not allow the observation of these children throughout their entire first year of life. We excluded children who died in the hospital in their first year of life. We used all diagnostic information (main and secondary diagnoses) from all inpatient episodes within the first year of life, or up to the RSV episode for those patients who were admitted to hospital with an RSV main or secondary diagnosis.

We reported odds ratios (OR) and 95% confidence intervals (CI) for each risk factor.

## Results

There were 39,863 hospitalised cases with an RSV main diagnosis between 2003 and 2021. Over the whole period, 70.7% (n = 28,162) of these hospitalisations were in infants. This proportion was slightly lower in recent years (average 2016–21: 65.6%). Among infant RSV inpatients, the mean age was 118 days (median: 85; first quartile: 43; third quartile: 178). Among adult RSV inpatients aged 18 years or older, the mean age was 74 years (median: 77; first quartile: 66; third quartile: 85). The percentage of males in all RSV main diagnosis hospitalisations between 2003 and 2021 was 54.8%, with 45.2% females.

### Hospitalisation numbers and rates in paediatric and adult patients

There was an increase in the number of RSV main diagnosis hospitalisations over time between the 2003/04 (n = 1,024) and 2019/20 (n = 2,487) respiratory years.

**Figure 1:**
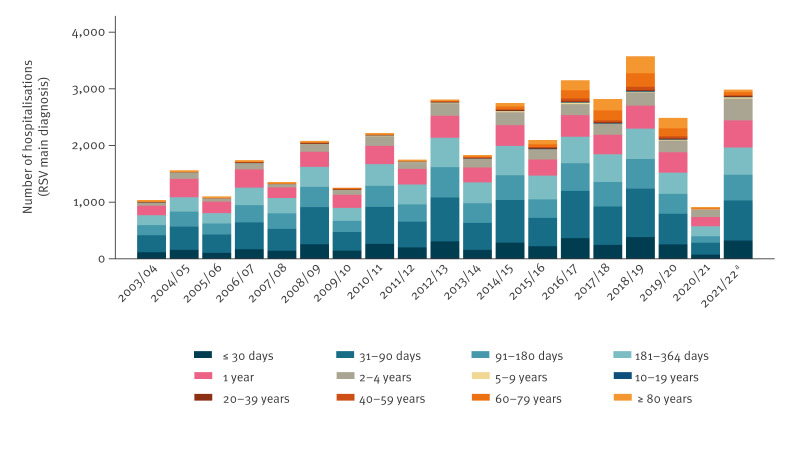
Number of RSV main diagnosis hospitalisations in paediatric and adult populations by age group, Switzerland, 2003/04–2021/22 (n = 39,382)

Figure 1 shows the absolute number of RSV hospitalisations by age group and year. The long-term trend of hospitalisations followed a biannual pattern. Respiratory syncytial virus respiratory years with a high number of hospitalisations were followed by RSV respiratory years with lower numbers. This pattern was interrupted by the COVID-19 pandemic in early 2020. There were 3,575 hospitalisations in 2018/19 and 2,487 hospitalisations in 2019/20, before we observed a substantial decrease in the number of cases in 2020/21 (n = 902) (Table 1). In July–December 2021 (for which we have data), there were 2,985 RSV main diagnosis hospitalisations. The RSV hospitalisations in adults, especially in patients aged 60 years or older, only increased in recent years (from 2016).

RSV: respiratory syncytial virus.

^a^ The numbers for 2021/22 refer to the period from July to December 2021 only.

The periods refer to respiratory years running from July to June the following year.

**Table 1:** Number of RSV main diagnosis hospitalisations and implied hospitalisation rates in paediatric and adult populations by age group, Switzerland, 2018/19–2020/21 (n = 6,964)

Age group	RSV main diagnosis hospitalisation						Hospitalisation rate per 1,000 population		
	2018/19		2019/20		2020/21		2018/19	2019/20	2020/21
	n	%	n	%	n	%			
< 1 year	2,300	64	1,524	61	576	64	26.7	18.0	6.8
0–30 days	385	11	256	10	75	8	53.6	36.4	10.7
31–90 days	856	24	540	22	210	23	59.6	38.3	14.9
91–180 days	523	15	353	14	115	13	24.3	16.7	5.5
181–364 days	536	15	375	15	176	20	12.4	8.9	4.2
1 year	406	11	358	14	164	18	4.7	4.1	1.9
2–4 years	232	6	207	8	125	14	0.9	0.8	0.5
5–59 years	101	3	75	3	23	3	0.0	0.0	0.0
≥ 60 years	536	15	323	13	14	2	0.3	0.2	0.0
**All ages**	**3,575**	**100**	**2,487**	**100**	**902**	**100**	**0.4**	**0.3**	**0.1**

RSV: respiratory syncytial virus.

**Figure 2:**
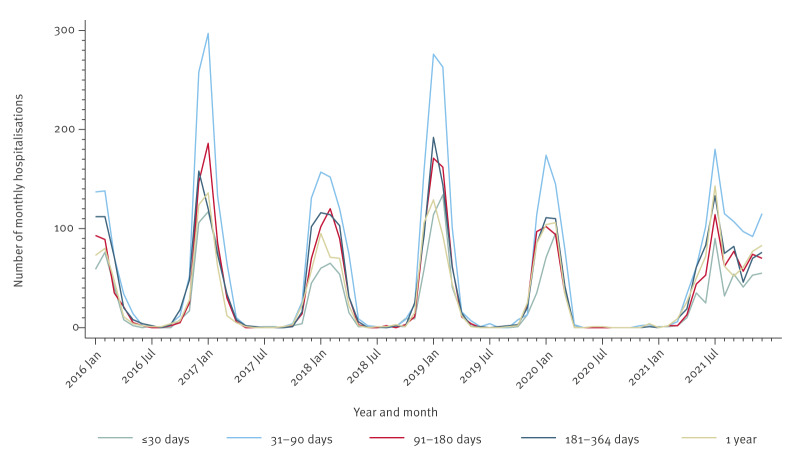
Number of RSV main diagnosis hospitalisations in cases aged under 2 years by age groups, Switzerland, 2016–2021 (n = 13,871)

The biannual trend was also observed for specific age groups, with the highest number of hospitalisations being observed in children aged 31 to 90 days, followed by those aged 91 to 180 days (Figure 2). Figure 2 shows monthly hospitalisation numbers between 2016 and 2021 for patients aged less than 2 years. Supplementary Figure S1 shows the monthly RSV hospitalisations in all age groups between 2003 and 2021. Supplementary Figure S2 shows the number of RSV hospitalisations per calendar year (2003–21) and by age groups.

RSV: respiratory syncytial virus.

Table 1 shows the number of RSV main diagnosis hospitalisations per 1,000 population by year for the three most recent RSV respiratory years in our data (2018/19–2020/21). The proportions of total hospitalisations show that the highest burden was observed in infants and adults aged ≥ 60 years.

### RSV-associated inpatient healthcare resource use and medical costs in paediatric and adult patients

Mean LOS of inpatients with an RSV main diagnosis between 2016 and 2021 decreased with increasing age in children, e.g. 5.5 days in 0–30-day-olds and 3.7 days in 1-year-olds. The number and proportion of RSV cases in the ICU were highest in the first 3 months of life; 18.0% (332/1,843) of 0–30-day-olds and 10.0% (423/4,219) of 31–90-day-olds needed ICU treatment. Many ICU patients needed ventilation, with proportions up to 64.8% (274/423) in the 31–90-day-olds. Supplementary Table S1 shows, for each age group, the summary statistics for LOS, proportion of ICU stay and ventilation, as well as length of ICU stay and ventilation.

Total yearly medical costs of hospitalisations with an RSV main diagnosis ranged between CHF 16.7 million (95% CI: 15.1–18.3; EUR 15.4 million) in 2020 and CHF 31.5 million (95% CI: 29.4–33.7; EUR 29.1 million) in 2019.

Overall mean medical costs per RSV main diagnosis hospitalisation across all age groups were estimated at CHF 8,698 (first quartile: 5,900, third quartile: 7,900; EUR 8,046). Mean medical costs were slightly lower in infants (CHF 8,458; EUR 7,824). Intensive care unit admission substantially increased the mean medical costs per hospitalisation, e.g. in infants without ICU admission the mean was estimated at CHF 6,479 (EUR 5,994) and with ICU admission at CHF 30,161 (EUR 27,901). Supplementary Table S2 shows for each age group the mean, median and IQR for the medical cost per case, by ICU status. Supplementary Table S3 shows for each age group the total medical costs and the mean, median and IQR for the cost per case, by year (2016–21) and for cases with an RSV main diagnosis and RSV secondary diagnosis.

### Proportion of RSV and other acute respiratory infection hospitalisations

**Figure 3:**
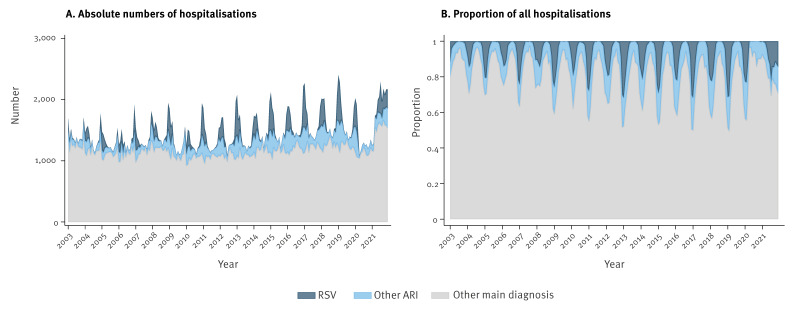
Number of infant hospitalisations per month with RSV main diagnosis, other acute respiratory infections or other main diagnosis, and proportion of total hospitalisations, Switzerland, 2003–2021 (n = 336,610)

ARI: acute respiratory infections; RSV: respiratory syncytial virus.

Between 2003 and 2021, 0.15% (n = 39,863) of all hospitalisations (n = 26,721,924) in all age groups had an RSV main diagnosis, and 2.3% (n = 616,185) had another ARI main diagnosis. Figure 3 shows the total number of hospitalisations by main diagnosis in infants, excluding hospitalisations at birth, over the course of 19 years. Between 2003 and 2021, 8.4% (n = 28,162) of all hospitalisations in infants (n = 336,610) were RSV-related and 12.2% (n = 40,946) had another ARI main diagnosis. The rates were higher in recent years, e.g. 11.0% RSV and 10.3% other ARI in 2021. Up to 50% of all infant hospitalisations were RSV and other ARI cases at the peak of the respiratory season. For instance, there were 703 RSV, 448 other ARI, and 1,132 other main diagnosis cases in February 2019, corresponding to 30.8%, 19.6% and 49.6% of all hospitalisations.

The figure shows number of hospitalisations in infants (age < 1 year), excluding hospitalisation at birth. The labels on the horizontal axis refer to January of that year. There were 28,162 RSV hospitalisations, 40,649 other ARI hospitalisations, and 267,502 hospitalisations with another diagnosis.

The proportion of RSV and ARI main diagnoses in paediatric patients (up to 18 years of age) between 2003 and 2021 was 9.2% in total (1.9%; n = 37,463 RSV and 7.3%; n = 140,759 ARI) and reached its maximum in January (14.8%; 5.6% RSV and 9.2% ARI).

In addition to RSV main diagnosis hospitalisations, there was a remarkable number of RSV secondary diagnosis hospitalisations. For instance, there were 341 RSV secondary diagnosis hospitalisations in addition to the 2,300 RSV main diagnosis hospitalisations in infants in the RSV respiratory year 2018/19; 50.7% (n = 173) of these RSV secondary diagnosis hospitalisations had another ARI main diagnosis. The numbers for the five most recent RSV respiratory years and by age group are provided in Supplementary Table S4.

### Characteristics of infant RSV inpatients

Of all infants hospitalised with an RSV main diagnosis between 2016 and 2021 (n =  9,473; only including infants with complete birth data), 7.1% (n = 673) were born before 35 gestational weeks (proportion in all newborns: 3.0%; n = 15,309) and 14.1% (n = 1,338) were born before week 37 (proportion in all newborns: 7.3%; n = 37,599). The proportion of those born before week 29 was 1.3% (n = 119; proportion in all newborns: 0.6%; n = 3,313). Between 2016 and 2021, 90.8% (8,598/9,473) of all infants hospitalised with RSV main diagnosis and 79.2% (605/764) of infants hospitalised with RSV main diagnosis and treated in ICU were born after gestational week 35 and without any diagnostic code for bronchopulmonary dysplasia (BPD) or congenital heart disease in a prior hospital stay. Supplementary Table S5 shows the number of patients under 2 years hospitalised with RSV and the proportion of patients with BPD or congenital heart disease by gestational age. Of all infants with an RSV main or secondary diagnosis between 2016 and 2019 (n = 8,356), 67.6% (n = 5,648) were born between October and March, 14.9% (n = 1,249) were born in December, 13.7% (n = 1,145) in November and 12.6% (n = 1,053) in January. Supplementary Figure S3 shows the proportion of infants hospitalised with RSV by month of birth.

**Figure 4:**
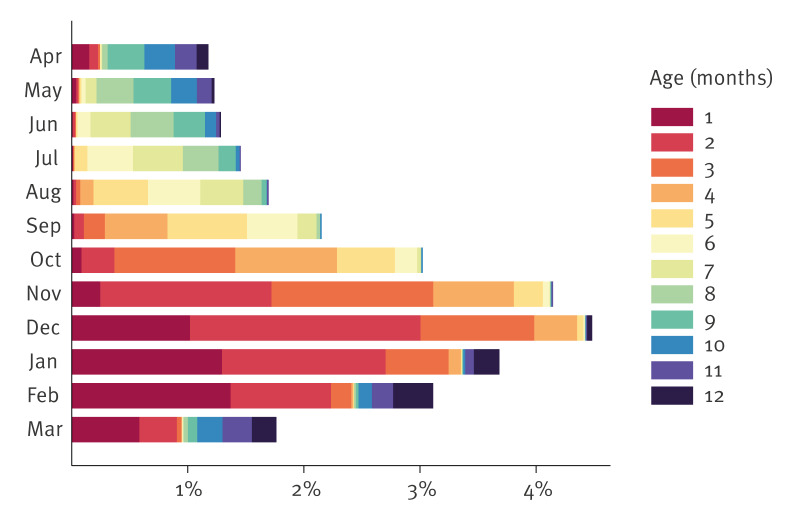
Proportion of RSV main or secondary hospitalisations in infants born between 2016*–*2019 by age and birth month, Switzerland (n = 348,002)

Figure 4 shows the percentage of infants by month of birth and age at hospitalisation. All births between 2016 and 2019 were pooled. The proportion of infants hospitalised within the first 6 months was substantially higher in those born between October and February. The cumulative proportions for each month by birth month are also provided in Supplementary Table S6.

RSV: respiratory syncytial virus.

The figure shows the proportion of infants (< 1 year) hospitalised with RSV by month of birth, by age at the time of hospitalisation. All births between 2016 and 2019 were pooled. For example, about 4.5% of infants born in December are hospitalised with RSV within the first year of life, and most of them are 1 or 2 months old (red bars) at the time of the hospitalisation. Proportions were calculated by dividing the number of RSV cases in HospReg by birth month by the total number of births in HospReg in the same month.

Between 2016 and 2021, there were 70 in-hospital deaths in patients with an RSV main diagnosis, corresponding to 0.4% of all cases with an RSV main diagnosis. Of all deaths with a code RSV, 66 (94.8%) were in patients aged 60 or older, and there were no deaths in infants. Cause of death is not reported in HospReg.

### Risk factors for RSV hospitalisation in infants

The results of the risk factor analysis are shown in Table 2. The list of risk factors is provided in Supplementary Table S7, along with the ICD-10-GM codes used to identify them in the data.

**Table 2:** Risk factor analysis for RSV hospitalisation in infants, Switzerland, 2012–2021 (n = 767,774)

Characteristics	OR	95% CI
**Year of birth **		
2012	1	1–1
2013	0.607***	0.561–0.656
2014	0.900**	0.840–0.965
2015	0.662***	0.614–0.714
2016	1.085*	1.016–1.159
2017	0.822***	0.766–0.882
2018	1.132***	1.060–1.209
2019	0.788***	0.733–0.847
2020	0.383***	0.350–0.419
**Quarter of birth **		
Q1 (Jan–Mar)	1	1–1
Q2 (Apr–Jun)	0.481***	0.453–0.510
Q3 (Jul–Sep)	0.760***	0.722–0.800
Q4 (Oct–Dec)	1.596***	1.527–1.668
**Birth weight **		
< 2,000 g	1.324***	1.157–1.516
2,000–2,499 g	1.256***	1.146–1.376
2,500–2,999 g	1.085**	1.033–1.140
≥ 3,000 g	1	1–1
**Gestational age **		
< 29 weeks	2.181***	1.718–2.769
29–31 weeks	2.259***	1.877–2.718
32–36 weeks	1.531***	1.420–1.651
≥ 37 weeks	1	1–1
Unknown	0.769	0.586–1.008
**Birth characteristic**		
Single birth	1	1–1
Multiple birth	1.284***	1.186–1.391
**Sex**		
Male	1	1–1
Female	0.864***	0.834–0.895
**Mother’s age at birth **		
< 25 years	1	1–1
25–34 years	0.894***	0.837–0.955
≥ 35 years	0.845***	0.788–0.906
Unknown	1.522	0.619–3.741
**Medical conditions **		
Congenital malformation of the great vessels	1.403***	1.177–1.671
Congenital malformation of the heart	1.814***	1.583–2.079
Congenital malformations of the respiratory system	2.071***	1.597–2.686
Congenital defect originating in perinatal period	1.139	0.996–1.304
Biliary atresia	3.954*	1.080–14.47
Haemophilia	1.917***	1.508–2.439
Bronchopulmonary dysplasia	1.250	0.971–1.608
Down’s syndrome	2.672***	1.983–3.602
Disorder of newborn related to slow fetal growth and fetal malnutrition	0.985	0.917–1.059
Immunodeficiency	1.593	0.662–3.831
Liver disease	1.173	0.391–3.516
Nervous system diseases	2.343***	1.885–2.912
Renal failure	1.483	0.741–2.966
Respiratory and cardiovascular disorder specific to the perinatal period	1.164***	1.103–1.228
Vitamin D deficiency	1.399	0.805–2.431
Cerebral palsy	1.209	0.596–2.452
Cystic fibrosis	5.036***	2.666–9.516

CI: confidence intervals; OR: odds ratio; RSV: respiratory syncytial virus.

Significance is indicated by asterisks * p < 0.05, ** p < 0.01, *** p < 0.001.

The population is all infants (< 1 year) born in hospital between 2012 and 2020 who did not die in hospital in first year of life. All available diagnostic information was used, i.e. all diagnoses coded at birth as well as in subsequent inpatient stays before RSV hospitalisation. Dependent variable: RSV diagnosis (main or secondary diagnosis) in first year of life.

The ORs for the birth years reflect the biannual variation. The time of birth has an important influence on the risk of admission with highest risk for those born in the last quarter of the year between October and December (OR: 1.596). Lower birth weight and gestational age are also important risk factors for RSV admission with a birth weight below 2,000 g resulting in an OR of 1.324 and being born before 29 weeks of gestation resulting in an OR of 2.181. Finally, male sex was associated with a higher risk of admission.

In terms of underlying medical conditions, 9 of the 17 pre-defined conditions were significantly associated with a higher risk for RSV hospitalisation with OR > 1.0. Conditions like cystic fibrosis, congenital malformations of the respiratory system, biliary atresia, nervous system diseases and Down’s syndrome had the highest OR. Note that for some of the medical conditions, the number of prevalent patients was very low, e.g. cystic fibrosis (n = 11 in RSV infants; n = 107 in non-RSV infants), cerebral palsy (n = 9; 145), renal failure (n = 9; 147), liver disease (n = 5; 88), immunodeficiency (n = 6; 103), or biliary atresia (n = 4; 25). The small number of observations may lead to wider CI.

Summary statistics for all potential explanatory variables for both groups (‘RSV’, ‘no RSV’) and results for alternative specifications are provided in Supplementary Tables S8 and S9.

## Discussion

This is the first study, to our knowledge, to investigate the characteristics of RSV inpatients and the resource use associated with inpatient treatment for RSV in Switzerland, and to assess the risk factors for RSV hospitalisation in a large population of infants. This study showed the substantial burden of RSV in inpatient care in terms of the number of hospitalisations and peaks in hospital capacity utilisation. Infants accounted for about two thirds of all hospitalisations with an RSV main diagnosis. Most hospitalised infants were born at term and did not have any pre-existing medical condition. This finding is in line with previous research from other countries [5,20,21,25] and shows that the highest burden of disease is in healthy infants in their first year of life. Respiratory syncytial virus hospitalisations in the adult population occurred mostly in individuals aged 60 years and above, but there were almost no cases in this population before 2016. Potential reasons for that are improved detection, better coding or a combination of both.

Our long-term epidemiological analysis of the number of RSV hospitalisations confirmed the biannual pattern previously shown in Switzerland and Germany [12,26,27]. We observed a trend of increasing numbers of inpatients and a recent increase in case numbers in both paediatric and adult patients. Reasons for this remain speculative and may be driven by population growth but also by improved coding or increased testing. We observed a substantial decrease in RSV hospitalisations in the period July 2020 to June 2021, which was a likely consequence of the prevention measures during the COVID-19 pandemic. The subsequent increase in hospitalisation numbers between July and December 2021 may have been driven by the high number of out-of-season infections, the increased availability of multiplex PCR testing, as well as a higher awareness for RSV after the experiences in the pandemic. Our estimates of the RSV hospitalisation rates before the start of the COVID-19 pandemic in 2020 were similar to those reported in other countries or regions. For example, the RSV hospitalisation rate of 26.7/1,000 infants in 2018/19 closely matches results from Denmark (years 2010–15; 29.4/1,000) [28], England (2007–08; 24.2/1,000) [5] or Portugal (2015/16–2017/18; 23.8/1,000) [20]. Our analysis shows that rates were highest in children under the age of 3 months, similar to studies from Denmark and Portugal. The same pattern was found in the United States (US), although with lower rates in all age groups [7,8].

Overall, 7.2% of all RSV hospitalisations between 2016 and 2021 involved ICU treatment, similar to results from Germany (7.0% in 2013–18 [6]). As expected, this proportion was substantially higher in younger age groups, with up to 18.0% in infants up to 30 days of age. In line with previous research, inpatient stays were longer in younger infants [20,28].

To our knowledge, there are no up-to-date estimates of the medical costs of RSV hospitalisations in Switzerland. A comparison of the cost estimates with results from other countries is difficult because of different RSV case definitions, study periods, or sample definitions, e.g. with respect to age groups. Using similar administrative hospital data and identifying RSV cases based on ICD-10-GM codes, a recent study from Germany reported mean costs of EUR 3,429 for the period 2010–19 [29]. This is ca 43% of our estimate of EUR 8,046, while per capita healthcare costs in Germany were around 58% of the per capita medical costs in Switzerland in 2020 [30]. In the US, where nominal per capita costs were 14% higher, mean costs per RSV hospitalisation in children under 5 years old were estimated at USD 13,596 (2016–19) (EUR 12,153 in 2019) [31]. In the same population, a study from Portugal estimated mean medical costs per hospitalisation of EUR 1,004 in 2018 [20], equal to 13% of the overall Swiss estimate (with Portugal showing per capita medical costs 77% below Switzerland). 

The risk factor analysis showed that low birth weight (in particular < 2,000 g) was associated with a higher risk of RSV hospitalisation, and this association was robust even after controlling for the presence of medical conditions. This result is in line with previous studies from Spain and Germany [3,6]. The study from Spain also found a higher risk for RSV hospitalisation in children whose mothers were below age 25 years at delivery, consistent with our results [3]. We were able to confirm the finding that male sex is associated with a substantially higher risk for RSV hospitalisation [25,32]. Gestational age below 37 weeks and low birth weight were also among the factors identified in a meta-analysis as having a significant effect on the risk for RSV infection, i.e. including not only studies assessing the hospitalisation risk [4]. The results from the risk factor analysis should be interpreted with caution. While the birth-related risk factors were known for all newborns, information about medical conditions was only present if they were coded at birth or in a subsequent inpatient stay. We therefore had more information about the additional medical conditions of patients treated in hospital multiple times, which might distort the effects of risk factors for an RSV hospitalisation.

There has been remarkable progress in the development of preventive interventions (vaccines and monoclonal antibodies) targeting paediatric [33] and adult populations [34] recently. Monoclonal antibodies and maternal immunisation are expected to reduce the burden of RSV in infants, especially with respect to hospitalisations [33]. As there are currently no vaccines against RSV available for children, several countries have recently approved and started to administer the monoclonal antibody nirsevimab for RSV prophylaxis in infants [35]. It is likely that a large proportion of RSV-related hospitalisations can be avoided in the next few years once the newly approved substances are widely administered. Early evidence from several European countries and the US suggests that nirsevimab may be highly effective in reducing RSV-related hospitalisations in real-world settings, with effectiveness estimates of 70–90% [36-38]. A Swiss expert working group with representatives from the relevant medical societies and the Swiss National Immunization Technical Advisory Group recently recommended that all infants should receive a single dose of nirsevimab, under the condition that it is covered by the mandatory health insurance [39]. In Switzerland, high-risk infants with BPD or hemodynamically significant heart disease are eligible for prophylaxis with the monoclonal antibody palivizumab, which requires monthly administration [40]. Our findings may be informative for other countries with a similar seasonality to Switzerland and may guide implementation of novel RSV prophylactic options such as long-acting monoclonal antibodies or vaccines. For countries with a similar population structure and healthcare system, at-risk groups might be similar but overall, the cost calculations will vary and hence, we encourage every country to perform a similar analysis.

This study has several limitations. Firstly, it is a retrospective analysis based on data that were originally not collected for research purposes. Therefore, some of the data, e.g. on diagnosis, may be incomplete. Secondly, some potentially relevant information for our analysis was not available in HospReg data. Examples are detailed information about inpatient HCRU such as in-hospital medications (including palivizumab) or data on more risk factors, e.g. socioeconomic and behavioural factors. Finally, HospReg only covers inpatient care. Consequently, we did not observe RSV-related HCRU and treatments for potential risk factors in other settings such as outpatient physician visits or emergency care units. In addition, as we only included inpatient cases with a coded RSV main diagnosis for the calculation of hospitalisation rates, we are likely to underestimate the true rates.

## Conclusion

The burden of RSV in inpatient care in Switzerland is substantial in terms of the number of hospitalisations and peaks in hospital capacity utilisation. While certain risk factors exist, most infants hospitalised with RSV do not have any known risk factor and the highest burden is observed in children born at term and without any underlying medical condition. Measures to protect all infants from an RSV hospitalisation are essential. The results from this study may be useful as inputs to health economic evaluations of preventive measures against RSV, such as vaccines or monoclonal antibodies, and to inform on the timing and coverage of these measures.

## Supplementary Data

583000 Supplement
