# Supplementary material for: Inpatient burden of respiratory syncytial virus (RSV) in Switzerland, 2003 to 2021: an analysis of administrative data
Source: Euro Surveill. 2024 Sep 26;29(39):2400119. doi: 10.2807/1560-7917.ES.2024.29.39.2400119 (PMC11484346; doi:10.2807/1560-7917.ES.2024.29.39.2400119)
Supplement: Supplement [file 24-00119_STUCKI_Supplement.pdf]

Supplementary material

Article title: Inpatient burden of respiratory syncytial virus (RSV) in Switzerland, 2003 to 2021: an analysis of administrative data

This supplementary material is hosted by *Eurosurveillance* as supporting information alongside the article *Inpatient burden of respiratory syncytial virus (RSV) in Switzerland, 2003 to 2021: an analysis of administrative data*, on behalf of the authors, who remain responsible for the accuracy and appropriateness of the content. The same standards for ethics, copyright, attributions and permissions as for the article apply. Supplements are not edited by *Eurosurveillance* and the journal is not responsible for the maintenance of any links or email addresses provided therein.

1 Hospitalization numbers and rates

Figure S1: Number of monthly RSV main diagnosis hospitalizations (2003-2021, all age groups), m1=January

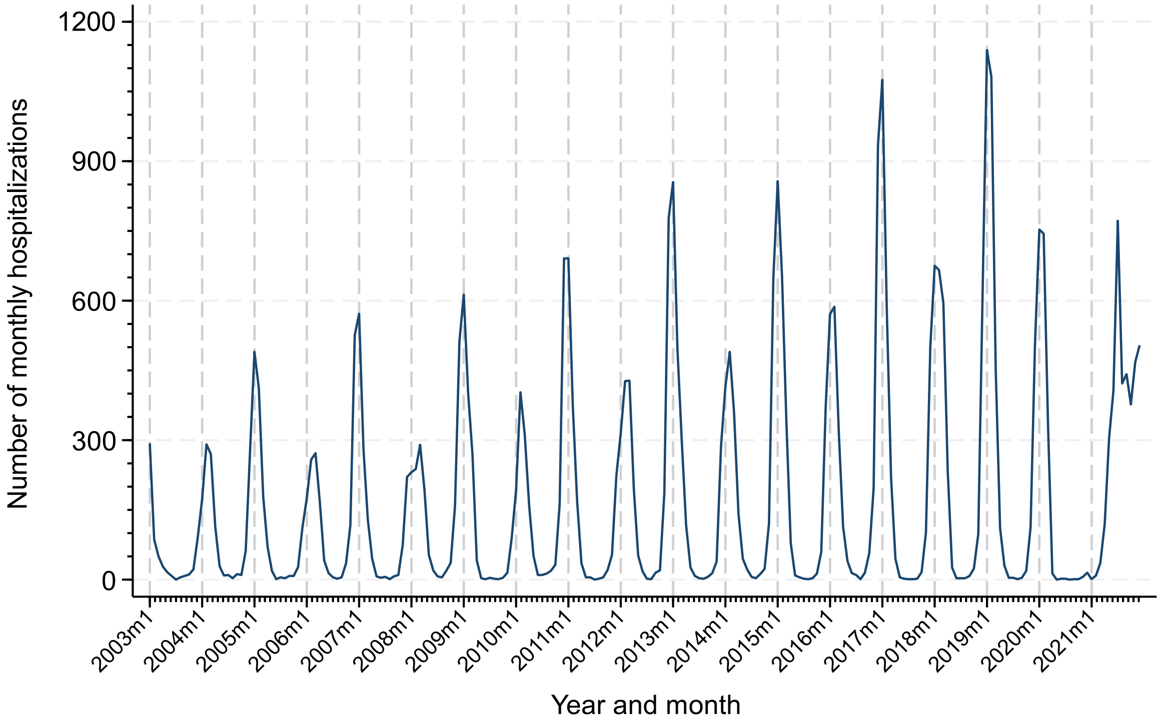

Inpatient burden of respiratory syncytial virus (RSV) in Switzerland, 2003 to 2021: an analysis of administrative data

Figure S2: Number of RSV main diagnosis hospitalizations by age groups and years (2003-2021)

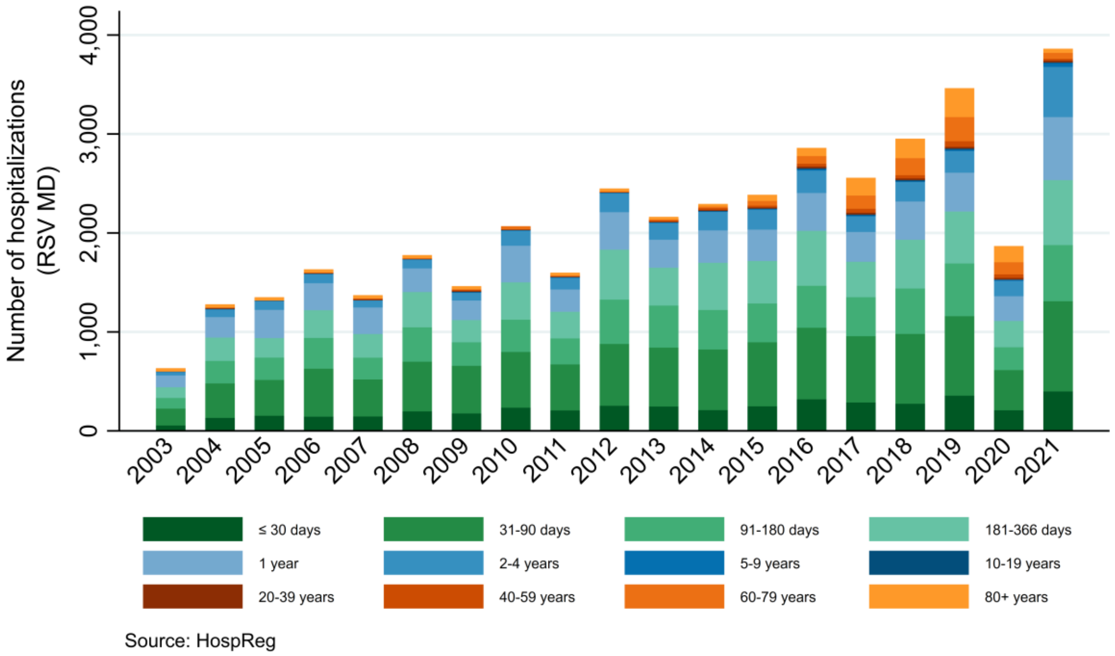

2 RSV inpatient health care resource use and medical costs

Table S1: Length of stay, ICU, and ventilation use of patients hospitalized with an RSV main diagnosis, by age group (2016-2021)

Age group	Length of stay (days)		Number (proportion) of patients in ICU	Length of ICU stay (hours)		Number (proportion) of patients in ICU with ventilation	Length of ventilation of patients in ICU (hours)	
	mean	SD	n (proportion)	mean	SD	n (proportion of all ICU)	mean	SD
≤30 days	5.5	4.0	332 (18.0%)	124	102	220 (66.3%)	107	91
31-90 days	4.4	3.2	423 (10.0%)	112	87	274 (64.8%)	102	76
91-180 days	4.1	3.3	119 (4.6%)	117	102	67 (56.3%)	110	107
181-366 days	3.8	3.1	89 (3.1%)	107	84	43 (48.3%)	110	85
1 year	3.7	3.9	86 (3.7%)	103	126	36 (41.2%)	95	122
2-4 years	3.7	3.0	48 (3.3%)	115	118	18 (37.5%)	149	145
5-19 years	3.8	3.0	7 (3.7%)	142	82	4 (57.1%)	95	82
20-59 years	7.0	6.5	27 (9.9%)	136	150	18 (66.7%)	119	147
60+ years	9.4	9.5	134 (7.6%)	96	106	86 (64.2%)	79	113

SD: Standard deviation; n: number; ICU: Intensive care unit

Table S2: Estimated medical costs for inpatients with RSV main diagnosis with/without ICU, mean, median and IQR per hospitalization, by age groups (2016-2021)

	**Estimated medical costs of inpatient RSV hospitalizations (assuming uniform base rate of CHF 10,000)**								
	**All RSV main diagnosis**			**RSV main diagnosis without ICU**			**RSV main diagnosis with ICU**		
	**Per case (CHF)**			**Per case (CHF)**			**Per case (CHF)**		
**Age group**	**Mean**	**Median**	**IQR**	**Mean**	**Median**	**IQR**	**Mean**	**Median**	**IQR**
<1 year	8458	6430	1010	6479	6430	620	30161	17264	31300
1 year	6848	5920	750	6129	5920	750	25757	11689	21400
2-4 years	7279	6050	670	6484	6050	670	30754	16205	32095
5-19 years	8360	6180	3210	7209	6180	2540	37967	27490	31670
20-59 years	13917	11380	11900	11518	11320	8630	35770	27400	37270
60+ years	13120	11380	9830	11839	11380	7910	28746	24105	11620

IQR: Inter-quartile range; CHF: Swiss Francs; ICU: Intensive care unit

Table S3: Estimated medical costs for inpatients with RSV main diagnosis, total and mean, median and IQR per hospitalization, by age groups and year

		**Estimated medical costs of inpatient RSV hospitalizations (assuming uniform base rate of CHF 10,000)**							
		**RSV main diagnosis**				**RSV secondary diagnosis & ARI main diagnosis**			
		**Total (mCHF)**	**Per case (CHF)**			**Total (mCHF)**	**Per case (CHF)**		
**Year**	**Age group**		**Mean**	**Median**	**IQR**		**Mean**	**Median**	**IQR**
2016	<1 year	17.0	8398	6430	960	1.5	6241	4450	3230
	1 year	2.8	7448	6470	1310	0.2	5189	6470	3720
	2-4 years	1.9	8587	6470	3000	0.4	8145	6710	4700
	5-19 years	0.3	9553	7970	3000	0.1	9430	6560	6650
	20-59 years	0.9	22847	18580	8807	0.1	9212	10730	6190
	60+ years	2.4	14658	12750	12560	0.7	10364	6710	7467
2017	<1 year	14.4	8419	6520	0	0.9	6132	4500	3980
	1 year	1.9	6452	6150	1110	0.2	5854	6310	3430
	2-4 years	1.1	7035	5800	1110	0.1	7015	6310	1346
	5-19 years	0.3	11347	6150	2540	0.1	5900	5005	2665
	20-59 years	0.7	13223	11800	14000	0.3	8485	8300	4700
	60+ years	4.5	14354	11800	13140	1.6	8916	8280	5860
2018	<1 year	16.6	8591	6450	0	0.8	5619	4870	3711
	1 year	2.8	7245	6050	130	0.4	10879	6320	4215
	2-4 years	1.9	9346	6050	3240	0.3	7280	6360	3680
	5-19 years	0.2	11245	6175	3110	0.2	10232	6360	5480
	20-59 years	0.5	11979	11320	9338	0.3	7946	6360	4290
	60+ years	4.6	12537	11320	10300	2.0	9592	8110	5000
2019	<1 year	19.4	8766	6290	1530	1.2	6937	4610	2790
	1 year	2.9	7324	6150	720	0.3	6119	5980	3300
	2-4 years	1.6	7383	6150	2470	0.4	7445	5980	2770
	5-19 years	0.2	7033	7025	2470	0.1	20317	5980	2250
	20-59 years	0.8	11393	11380	5230	0.4	8741	6290	3030
	60+ years	6.6	12371	11380	9480	2.6	8994	7950	5400
2020	<1 year	9.4	8437	6050	1530	0.4	5596	4610	2930
	1 year	1.6	6589	6050	0	0.2	8103	6260	1650
	2-4 years	1.1	7270	6050	2960	0.2	6869	5855	1650
	5-19 years	0.1	6246	6050	6120	0.1	8610	5660	10080
	20-59 years	0.6	13302	11530	12000	0.2	6292	6255	4510
	60+ years	3.9	13436	11530	8130	1.4	10150	8430	5280
2021	<1 year	20.7	8173	5900	0	0.8	4648	4530	3060
	1 year	4.0	6242	5900	0	0.7	8113	5900	3520
	2-4 years	3.0	5920	5900	280	0.5	6053	6000	1650
	5-19 years	0.4	6688	6040	280	0.1	7703	6000	3520
	20-59 years	0.3	12576	11020	3660	0.1	6174	6000	4130
	60+ years	1.2	12063	11020	7180	0.4	11420	8570	8480

IQR: Inter-quartile range; CHF: Swiss Francs

3 Coding of RSV

Table S4: Coding of RSV as main diagnosis and secondary diagnosis in HospReg, by RSV respiratory year (July-June 2015/16-2020/21)

		**RSV respiratory year (July-June)**					
	**Age group**	**2015/2016**	**2016/2017**	**2017/2018**	**2018/2019**	**2019/2020**	**2020/2021**
**Number of cases with RSV main diagnosis**	<1 year	1470	2157	1846	2300	1524	576
	1 year	285	381	345	406	358	164
	2-4 years	179	194	189	232	207	125
	5+ years	162	419	440	637	398	37
**Number of cases with RSV secondary diagnosis**	<1 year	251	351	288	341	203	70
	1 year	59	97	81	109	55	40
	2-4 years	67	77	70	87	89	49
	5+ years	268	836	844	1274	680	46
**Number (proportion) of cases with ARI main diagnosis among RSV secondary diagnosis cases**	<1 year	161 (64%)	208 (59%)	149 (52%)	173 (51%)	100 (49%)	33 (47%)
	1 year	28 (48%)	46 (47%)	39 (48%)	52 (48%)	25 (45%)	19 (48%)
	2-4 years	34 (51%)	39 (51%)	31 (44%)	52 (60%)	38 (43%)	20 (41%)
	5+ years	56 (21%)	246 (29%)	251 (30%)	349 (27%)	190 (28%)	17 (37%)
**Number of cases with ARI main diagnosis**	<1 year	2882	2794	2989	3093	2384	1685
	1 year	2074	1859	2023	1898	1564	1358
	2-4 years	2509	2228	2400	2432	2059	1563
	5+ years	27198	32076	34503	32934	33448	45946

4 Characteristics of RSV inpatients

Table S5: Number of patients hospitalized with RSV main diagnosis (2016-2021) at age 0 (i.e., as infant) or age 1 year and proportion of patients with bronchopulmonary dysplasia (BPD) or congenital heart disease (CHD), by gestational age category

**Age at RSV hospitalization**	**Gestational age**	**Patients number**	**Proportion of total by age**	**Proportion of patients with BPD and/or CHD**
0	<29 weeks	119	1%	60%
0	29-31 weeks	161	2%	24%
0	32-36 weeks	1058	11%	5%
0	>=37 weeks	8120	86%	2%
0	unknown	15	0%	0%
**0**	**total**	**9473**	**100%**	**3%**
1	<29 weeks	64	4%	72%
1	29-31 weeks	49	3%	22%
1	32-36 weeks	180	10%	8%
1	>=37 weeks	1491	83%	3%
1	unknown	4	0%	25%
**1**	**total**	**1788**	**100%**	**7%**

Figure S3: Proportion of infants hospitalized with RSV (main or secondary diagnosis) and born between 2016-2019 by month of birth

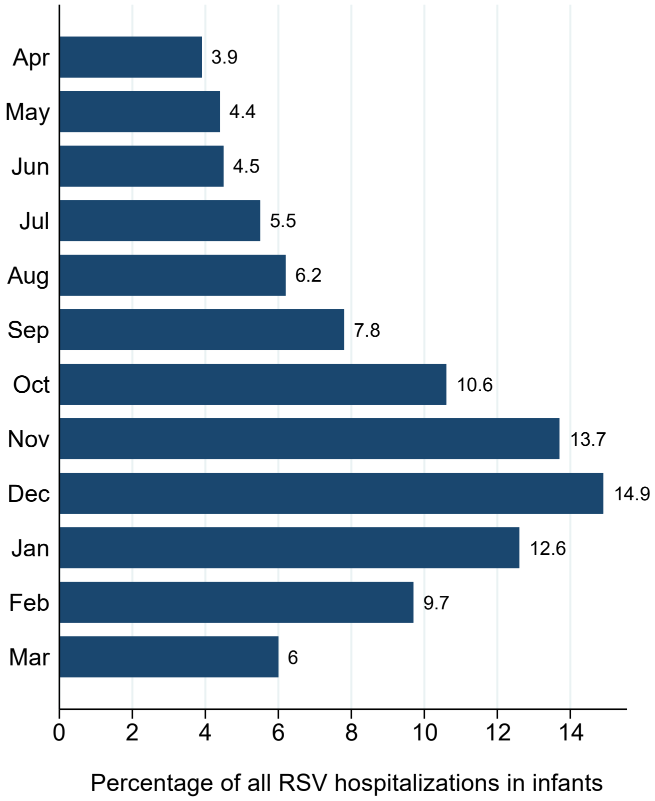

Table S6: Cumulative proportion (%) of newborns born between 2016-2019 hospitalized with RSV (main or secondary diagnosis) as infant, by age and month of birth

	**Proportion (%) of newborns that are hospitalized with RSV up to age in months**											
**Birth month**	**1**	**2**	**3**	**4**	**5**	**6**	**7**	**8**	**9**	**10**	**11**	**12**
Jan	1.3	2.7	3.2	3.4	3.4	3.4	3.4	3.4	3.4	3.4	3.5	3.7
Feb	1.4	2.2	2.4	2.4	2.4	2.4	2.4	2.4	2.5	2.6	2.8	3.1
Mar	0.6	0.9	1.0	1.0	1.0	1.0	1.0	1.0	1.1	1.3	1.6	1.8
Apr	0.2	0.2	0.2	0.2	0.3	0.3	0.3	0.3	0.6	0.9	1.1	1.2
May	0.0	0.1	0.1	0.1	0.1	0.1	0.2	0.5	0.9	1.1	1.2	1.2
Jun	0.0	0.0	0.0	0.0	0.1	0.2	0.5	0.9	1.1	1.2	1.3	1.3
Jul	0.0	0.0	0.0	0.0	0.1	0.5	1.0	1.3	1.4	1.4	1.5	1.5
Aug	0.0	0.0	0.1	0.2	0.7	1.1	1.5	1.6	1.7	1.7	1.7	1.7
Sep	0.0	0.1	0.3	0.8	1.5	1.9	2.1	2.1	2.1	2.1	2.1	2.1
Oct	0.1	0.4	1.4	2.3	2.8	3.0	3.0	3.0	3.0	3.0	3.0	3.0
Nov	0.2	1.7	3.1	3.8	4.1	4.1	4.1	4.1	4.1	4.1	4.1	4.1
Dec	1.0	3.0	4.0	4.4	4.4	4.4	4.4	4.4	4.4	4.4	4.4	4.5

5 Risk factors for RSV hospitalization in infants

5.1 List of risk factors

Table S7: Medical conditions considered as potential risk factors for RSV and their operationalization (ICD-10-GM codes)

Risk factor	ICD-10-GM codes	Organ system	References
Congenital malformation of the great vessels	Q25, Q26	Cardiovascular	[1]
Congenital malformation of the heart	Q20-Q24	Cardiovascular	[2, 3]
Congenital malformations of the respiratory system	Q30-Q34	Respiratory	[1]
Congenital defect originating in perinatal period	Q02, Q35, Q36, Q37, Q44, Q60, Q61, P70.0, P70.1, P70.2, P78.8	Other congenital	[1]
Biliary atresia	Q44.2	Other	[4]
Haemophilia	D51-D53, D65-D68, D50.0, D50.8, D50.9, D69.1, D69.3, D69.4, D69.5, D69.6	Haematological	[1]
Bronchopulmonary dysplasia	P27.1	Respiratory	[5, 6]
Down Syndrome	Q90	Down	[1, 2, 7]
Disorder of newborn related to slow fetal growth and fetal malnutrition	P05	Intrauterine growth retardation	[1]
Immunodeficiency	D80-D90	Immunological	[2]
Liver disease	B18, I85, I86, I98, K70, K72, K74, K71.1, K71.3, K71.5, K71.7, K76.0, K76.2, K76.9, Z94.4	Other	[1]
Nervous system diseases	G10-G13, G20, G22, G32, G35, G37, G40, G41, R56, G25.4, G25.5, G31.2, G31.8, G31.9, G93.1, G93.4, R47.0	Neurological	[1, 2]
Renal failure	N18, N19, N25, I12.0, I13.1, Z49.0, Z49.2, Z94.0, Z99.2	Other	[1]
Sickle cell anaemia	D57	Haematological	[1]
Respiratory and cardiovascular disorder specific to the perinatal period	P20-P26, P28, P29, P27.0, P27.8, P27.9	Cardiovascular	[1]
Vitamin D deficiency	E55	Other	[1]
Cerebral palsy	G80-G83	Neurological	[2]
Cystic fibrosis	E84	Respiratory	[2]

ICD-10-GM: International Classification of Diseases, version 10, German modification

5.2 Summary statistics

Table S8 shows the mean and standard deviation (SD) for all explanatory variables, i.e., risk factors, in the two groups of patients, i.e., the newborns with a hospitalization with RSV in the first year of life ("RSV") and those without ("no RSV"). The last two columns show the group differences and whether these were statistically significant.

Table S8: Descriptive statistics of RSV and non-RSV population (all births in Swiss hospitals, 2012-2020). RSV case defined as hospitalization with RSV main or secondary diagnosis in first year of life

	RSV		no RSV			
	**mean**	**SD**	**mean**	**SD**	**diff in means**	**z**
**Year of birth**						
2012	0.130	0.336	0.107	0.309	-0.023***	(-8.384)
2013	0.080	0.271	0.108	0.310	0.028***	(10.284)
2014	0.121	0.327	0.110	0.313	-0.011***	(-3.944)
2015	0.091	0.288	0.113	0.316	0.021***	(7.705)
2016	0.151	0.358	0.114	0.317	-0.037***	(-13.326)
2017	0.114	0.318	0.113	0.317	-0.001	(-0.263)
2018	0.155	0.362	0.113	0.316	-0.042***	(-15.045)
2019	0.106	0.308	0.112	0.315	0.005	(1.866)
2020	0.052	0.221	0.111	0.314	0.059***	(21.489)
**Quarter of birth**						
Q1 (Jan-Mar)	0.252	0.434	0.240	0.427	-0.012**	(-3.265)
Q2 (Apr-Jun)	0.128	0.334	0.252	0.434	0.124***	(32.447)
Q3 (Jul-Sep)	0.215	0.411	0.268	0.443	0.053***	(13.667)
Q4 (Oct-Dec)	0.406	0.491	0.241	0.428	-0.165***	(-43.537)
**Birth weight**						
<2000g	0.057	0.231	0.020	0.139	-0.037***	(-29.666)
2000-2499g	0.068	0.251	0.042	0.201	-0.026***	(-14.371)
2500-2999g	0.190	0.392	0.180	0.384	-0.010**	(-2.905)
>=3000g	0.685	0.464	0.758	0.428	0.072***	(19.148)
**Gestational age**						
<29 weeks	0.013	0.112	0.003	0.054	-0.010***	(-19.595)
29-31 weeks	0.018	0.132	0.005	0.068	-0.013***	(-21.488)
32-36 weeks	0.113	0.316	0.060	0.238	-0.052***	(-24.784)
>=37 weeks	0.853	0.354	0.928	0.259	0.075***	(32.448)
unknown	0.004	0.065	0.005	0.068	0.000	(0.712)
**Multiple births**	0.070	0.255	0.035	0.184	-0.035***	(-21.384)
**Sex (female)**	0.450	0.498	0.487	0.500	0.037***	(8.337)
**Mother's age at birth**						
<25 years	0.079	0.270	0.069	0.254	-0.010***	(-4.479)
25-34 years	0.614	0.487	0.613	0.487	-0.000	(-0.110)
>=35 years	0.307	0.461	0.318	0.466	0.011**	(2.601)
unknown	0.000	0.020	0.000	0.014	-0.000	(-1.415)
**Number of organs affected from medical conditions**						
0	0.747	0.435	0.819	0.385	0.072***	(21.234)
1	0.089	0.285	0.072	0.258	-0.017***	(-7.532)
2	0.137	0.344	0.097	0.296	-0.040***	(-15.270)
3	0.024	0.153	0.011	0.103	-0.013***	(-14.528)
4 or more	0.003	0.050	0.001	0.028	-0.002***	(-6.720)
**Medical conditions**						
Congenital malformation of the great vessels	0.014	0.119	0.004	0.062	-0.010***	(-18.672)
Congenital malformation of the heart	0.024	0.153	0.008	0.086	-0.017***	(-21.263)
Congenital malformations of the respiratory system	0.005	0.070	0.002	0.039	-0.003***	(-9.643)
Congenital defect originating in perinatal period	0.018	0.133	0.012	0.107	-0.006***	(-6.861)
Biliary atresia	0.000	0.017	0.000	0.006	-0.000***	(-5.026)
Hemophilia	0.006	0.079	0.002	0.040	-0.005***	(-12.795)
Bronchopulmonary dysplasia	0.008	0.092	0.002	0.042	-0.007***	(-17.309)
Chronic circulatory diseases	0.000	0.012	0.000	0.007	-0.000	(-1.491)
Down Syndrome	0.004	0.064	0.001	0.028	-0.003***	(-12.865)
Disorder of newborn related to slow fetal growth and fetal malnutrition	0.080	0.271	0.069	0.254	-0.010***	(-4.535)
Immunodeficiency	0.000	0.021	0.000	0.012	-0.000**	(-3.062)
Liver disease	0.000	0.020	0.000	0.011	-0.000**	(-2.733)
Nervous system diseases	0.007	0.084	0.003	0.050	-0.005***	(-10.389)
Renal failure	0.001	0.026	0.000	0.014	-0.000***	(-3.918)
Sickle cell anemia	0.000	0.012	0.000	0.007	-0.000	(-1.608)
Respiratory and cardiovascular disorder specific to the perinatal period	0.152	0.359	0.104	0.305	-0.048***	(-17.759)
Vitamin D deficiency	0.001	0.035	0.000	0.020	-0.001***	(-4.450)
Cerebral palsy	0.001	0.026	0.000	0.014	-0.000***	(-3.965)
Cystic fibrosis	0.001	0.029	0.000	0.012	-0.001***	(-6.388)
**Observations**	**13102**		**754681**		**767783**	

** $p < 0.05$, ** $p < 0.01$, *** $p < 0.001$; Note: categorical variables are coded as indicator variables at the individual level, i.e., a mean of 0.0 represents a proportion of 0%, a mean of 1.0 represents a proportion of 100%*

5.3 Regression results

We estimated three different specifications for the multivariate risk factor model. The paper shows the results for specification 3.

1. Only birth-related factors (year of birth, quarter of birth (Q1-Q4), sex, birth weight, gestational age, siblings in same delivery (multiple birth), mother's age)
2. Birth-related factors and the number of organs affected by medical conditions
3. Birth-related factors and single medical conditions

The number of organs affected by medical conditions was obtained from classifying the selected single medical conditions by organ system.

Table S9: Odds ratios (95% confidence intervals) from logit regression. Dependent variable: RSV diagnosis (main or secondary diagnosis) in first year of life. Population: all newborns who did not die in hospital in first year of life

	**Model 1**	**Model 2**	**Model 3**
	**Only birth-related variables**	**Birth-related variables and number of organs affected by conditions**	**Birth-related variables and medical diagnoses**
**Year of birth**			
2012	1	1	1
	[1,1]	[1,1]	[1,1]
2013	0.606***	0.606***	0.607***
	[0.561,0.656]	[0.561,0.656]	[0.561,0.656]
2014	0.902**	0.900**	0.900**
	[0.841,0.967]	[0.840,0.965]	[0.840,0.965]
2015	0.665***	0.662***	0.662***
	[0.617,0.717]	[0.614,0.713]	[0.614,0.714]
2016	1.091**	1.081*	1.085*
	[1.022,1.166]	[1.012,1.155]	[1.016,1.159]
2017	0.828***	0.818***	0.822***
	[0.771,0.888]	[0.762,0.878]	[0.766,0.882]
2018	1.138***	1.123***	1.132***
	[1.066,1.216]	[1.051,1.199]	[1.060,1.209]
2019	0.795***	0.785***	0.788***
	[0.740,0.855]	[0.730,0.844]	[0.733,0.847]
2020	0.388***	0.382***	0.383***
	[0.355,0.425]	[0.349,0.418]	[0.350,0.419]
**Quarter of birth**			
Q1 (Jan-Mar)	1	1	1
	[1,1]	[1,1]	[1,1]
Q2 (Apr-Jun)	0.481***	0.481***	0.481***
	[0.453,0.510]	[0.453,0.510]	[0.453,0.510]
Q3 (Jul-Sep)	0.760***	0.759***	0.760***
	[0.722,0.800]	[0.722,0.799]	[0.722,0.800]
Q4 (Oct-Dec)	1.594***	1.592***	1.596***
	[1.526,1.666]	[1.524,1.664]	[1.527,1.668]
**Birth weight**			
<2000g	1.475***	1.182*	1.324***
	[1.300,1.675]	[1.034,1.351]	[1.157,1.516]
2000-2499g	1.296***	1.145**	1.256***

	[1.190,1.411]	[1.047,1.253]	[1.146,1.376]
2500-2999g	1.092***	1.036	1.085**
	[1.042,1.144]	[0.987,1.088]	[1.033,1.140]
>=3000g	1	1	1
	[1,1]	[1,1]	[1,1]
**Gestational age**			
<29 weeks	3.080***	3.067***	2.181***
	[2.522,3.762]	[2.505,3.754]	[1.718,2.769]
29-31 weeks	2.702***	2.687***	2.259***
	[2.262,3.227]	[2.243,3.218]	[1.877,2.718]
32-36 weeks	1.591***	1.620***	1.531***
	[1.479,1.711]	[1.505,1.745]	[1.420,1.651]
>=37 weeks	1	1	1
	[1,1]	[1,1]	[1,1]
unknown	0.801	0.800	0.769
	[0.612,1.050]	[0.611,1.049]	[0.586,1.008]
**Multiple birth**	1.259***	1.273***	1.284***
	[1.163,1.363]	[1.176,1.379]	[1.186,1.391]
**Sex (female)**	0.858***	0.866***	0.864***
	[0.828,0.889]	[0.836,0.897]	[0.834,0.895]
**Mother's age at birth**			
<25 years	1	1	1
	[1,1]	[1,1]	[1,1]
25-34 years	0.891***	0.895***	0.894***
	[0.834,0.951]	[0.838,0.956]	[0.837,0.955]
>=35 years	0.846***	0.850***	0.845***
	[0.789,0.907]	[0.793,0.911]	[0.788,0.906]
unknown	1.508	1.527	1.522
	[0.614,3.706]	[0.621,3.754]	[0.619,3.741]
**Number of organs affected from medical conditions**			
0		1	
		[1,1]	
1		1.243***	
		[1.162,1.329]	
2		1.234***	
		[1.168,1.304]	
3		1.681***	
		[1.488,1.899]	
4 or more		2.263***	
		[1.580,3.242]	
**Medical conditions**			
Congenital malformation of the great vessels			1.403***
			[1.177,1.671]
Congenital malformation of the heart			1.814***
			[1.583,2.079]
Congenital malformations of the respiratory system			2.071***
			[1.597,2.686]
Congenital defect originating in perinatal period			1.139
			[0.996,1.304]
Biliary atresia			3.954*
			[1.080,14.47]
Hemophilia			1.917***

			[1.508,2.439]
Bronchopulmonary dysplasia			1.250
			[0.971,1.608]
Down Syndrome			2.672***
			[1.983,3.602]
Disorder of newborn related to slow fetal growth and fetal malnutrition			0.985
			[0.917,1.059]
Immunodeficiency			1.593
			[0.662,3.831]
Liver disease			1.173
			[0.391,3.516]
Nervous system diseases			2.343***
			[1.885,2.912]
Renal failure			1.483
			[0.741,2.966]
Respiratory and cardiovascular disorder specific to the perinatal period			1.164***
			[1.103,1.228]
Vitamin D deficiency			1.399
			[0.805,2.431]
Cerebral palsy			1.209
			[0.596,2.452]
Cystic fibrosis			5.036***
			[2.666,9.516]
**Observations**	**767774**	**767774**	**767774**
**Pseudo R^2**	**0.0347**	**0.0357**	**0.0378**

*Exponentiated coefficients / Odds ratios; 95% CI in brackets; * $p < 0.05$, ** $p < 0.01$, *** $p < 0.001$*

6 References

1. Cai, W., et al., *Risk factors for hospitalized respiratory syncytial virus disease and its severe outcomes.* Influenza Other Respir Viruses, 2020. **14**(6): S. 658-670.
2. Murray, J., et al., *Risk factors for hospital admission with RSV bronchiolitis in England: a population-based birth cohort study.* PloS one, 2014. **9**(2): S. e89186.
3. Cilla, G., et al., *Risk factors for hospitalization due to respiratory syncytial virus infection among infants in the Basque Country, Spain.* Epidemiol Infect, 2006. **134**(3): S. 506-13.
4. Zachariah, P., M. Ruttenber, and E.A. Simões, *Hospitalizations due to respiratory syncytial virus in children with congenital malformations.* The Pediatric infectious disease journal, 2011. **30**(5): S. 442-445.
5. Gil-Prieto, R., et al., *Respiratory Syncytial Virus Bronchiolitis in Children up to 5 Years of Age in Spain: Epidemiology and Comorbidities: An Observational Study.* Medicine (Baltimore), 2015. **94**(21): S. e831.
6. Chaw, P.S., et al., *Respiratory syncytial virus-associated acute lower respiratory infections in children with bronchopulmonary dysplasia: systematic review and meta-analysis.* The Journal of infectious diseases, 2020. **222**(Supplement_7): S. S620-S627.
7. Bloemers, B.L., et al., *Down syndrome: a novel risk factor for respiratory syncytial virus bronchiolitis—a prospective birth-cohort study.* Pediatrics, 2007. **120**(4): S. e1076-e1081.
